# Cost-effectiveness analysis of digital therapeutics for home-based cardiac rehabilitation for patients with chronic heart failure: model development and data analysis

**DOI:** 10.1186/s12962-023-00489-x

**Published:** 2023-11-06

**Authors:** Tianyi Liu, Yiyang Zhan, Silei Chen, Wenhong Zhang, Jian Jia

**Affiliations:** 1 School of Business, Nanjing University, Nanjing, 210093 China; 2 Departments of Geriatric Practice, The First Affiliated Hospital of Nanjing Medical University, Nanjing, 210029 China; 3 Medical School, Nanjing University, Nanjing, China; 4 National Institute of Healthcare Data Science, Nanjing University, Nanjing, China; 5 Departments of General Practice, The First Affiliated Hospital of Nanjing Medical University, Nanjing, China

**Keywords:** Heart failure, Digital therapeutics, Cardiac rehabilitation, Home-based cardiac rehabilitation, Cost-effectiveness analysis, Markov model

## Abstract

**Background:**

In recent years, numerous guidelines and expert consensus have recommended the inclusion of digital technologies and products in cardiac rehabilitation. Digital therapeutics (DTx) is an evidence-based medicine that uses digital means for data collection and monitoring of indicators to control and optimize the treatment, management, and prevention of disease.

**Objective:**

This study collected and reviewed real-world data and built a model using health economics assessment methods to analyze the potential cost-effectiveness of DTx applied to home-based cardiac rehabilitation for patients with chronic heart failure. From the perspective of medical and health decision-makers, the economic value of DTx is evaluated prospectively to provide the basis and reference for the application decision and promotion of DTx.

**Methods:**

Markov models were constructed to simulate the outcomes of DTx for home-based cardiac rehabilitation (DT group) compared to conventional home-based cardiac rehabilitation (CH group) in patients with chronic heart failure. The model input parameters were clinical indicators and cost data. Outcome indicators were quality-adjusted life years (QALYs) and incremental cost-effectiveness ratios (ICERs). The robustness of the evaluation methods and results was tested using sensitivity analyses. Clinical indicators, cost data, and health utility values were obtained from real-world data, including clinical study data, published literature, and public website information.

**Results:**

The Markov model simulated a time span of 10 years, with a cycle set at one month, for 120 cycles. The results showed that the per capita cost of the CH group was 38,442.11 CNY/year, with a QALY of 0.7196 per person per year. The per capita cost of the DT group was 42,300.26 CNY/year, with a QALY of 0.81687 per person per year. The ICER per person was 39,663.5 CNY/QALY each year, which was below the willingness-to-pay threshold of 85,698 CNY (China's GDP per capita in 2022).

**Conclusions:**

DTx for home-based cardiac rehabilitation is an extremely cost-effective rehabilitation option compared with conventional home-based cardiac rehabilitation. DTx for home-based cardiac rehabilitation is potentially valuable from the perspective of healthcare decision-makers.

## Introduction

Heart failure (HF) is a cardiac circulation disorder caused by systolic and diastolic dysfunction of the heart, which results in blood pooling in the venous system and inadequate blood perfusion in the arterial system [1]. As a chronic disease with approximately 38 million patients worldwide and an annual economic burden of approximately $108 billion, chronic HF has made rehabilitation for HF a major public health concern [2–5]. *2021 ESC Guidelines for the Diagnosis and Treatment of Acute and Chronic Heart Failure*, the *2018 Chinese Guidelines for the Diagnosis and Treatment of Heart Failure*, and the *2020 Chinese Expert Consensus on Cardiac Rehabilitation in Chronic Heart Failure* have pointed out that, cardiac rehabilitation (CR) is recommended for the treatment of chronic HF [6–8]. According to *Guidelines for Cardiac Rehabilitation Programs by AACVPR*, CR is a specialty area of medical supervision that uses a multidisciplinary approach to implement a comprehensive secondary prevention program for cardiovascular disease [9]. It has been classified as a Class I recommendation for the treatment of cardiovascular disease by the European Society of Cardiology, the American Heart Association, and the American College of Cardiology [10]. CR has been shown to be effective in controlling cardiovascular disease risk factors, reducing the risk of cardiovascular disease, increasing patient compliance in establishing healthy lifestyle behaviors, and improving quality of life [9, 10]. It also promotes the continuity of care for patients with cardiovascular disease and effectively reduces the incidence of cardiovascular events, hospitalization, and mortality [11, 12]. CR specifically includes medical assessment, psychosocial assessment, exercise prescription, cardiac risk factor intervention, patient education, behavioral guidance, and clinical outcome assessment, and is managed in an integrated manner through five core prescriptions (pharmacological prescription, exercise prescription, nutritional prescription, psychological prescription, and smoking and alcohol cessation prescription) [9, 13]. Exercise is a safe and effective way to improve the quality of life and exercise capacity of patients with HF, and it can significantly reduce the risk of hospitalization and death [14]. Therefore, the core of CR is to provide patients with systematic exercise training and physical activity advice and develop scientifically sound exercise prescriptions that increase patients' daily physical activity to a level that promotes health, improves cardiopulmonary function, and reduces the risk of chronic disease [9, 13, 15].

**Fig. 1:**
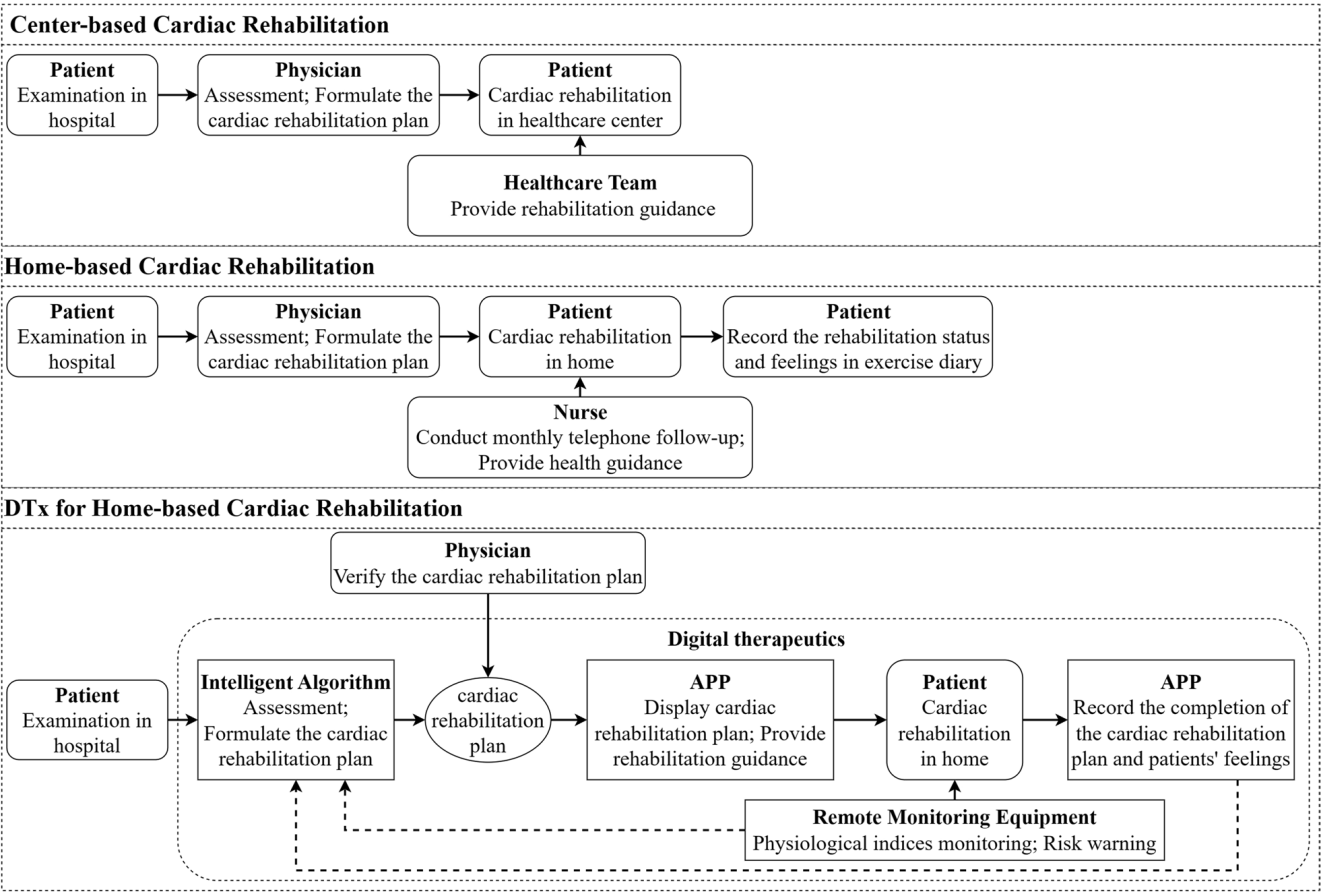
Ways of cardiac rehabilitation

Currently, there are two ways of CR for chronic HF: rehabilitation center-based CR and home-based CR (Fig. 1. Ways of cardiac rehabilitation).

In rehabilitation-center-based CR, patients visit a rehabilitation center and exercise using relevant medical equipment and devices with the assistance and guidance of a rehabilitation nurse. Center-based CR is very beneficial for the effective implementation of exercise prescriptions and the monitoring of feedback on exercise status. However, there are limitations to this rehabilitation program owing to the high costs, additional time, and transportation costs for the patient to travel to the rehabilitation center [16, 17]. In home-based CR, patients undergo cardiopulmonary function testing and assessment in the hospital, and the physician formulates an exercise prescription based on the results of the assessments. The patients exercise at home on their own according to the exercise prescription. Home-based CR significantly improves the quality of life and exercise capacity in patients with HF, and the patient benefit does not differ significantly from that of outpatient rehabilitation [18–21]. Home-based CR is effective in avoiding barriers to medical care and reducing risks of exposure to infectious diseases for specific reasons, such as the coronavirus disease 2019 (COVID-19) pandemic [22, 23]. In addition, home-based CR reduces the difficulty in accessing medical care for patients with limited mobility. Patients with chronic HF prefer home-based CR over rehabilitation center-based CR [24, 25]. Therefore, home-based CR has advantages in terms of medical accessibility and affordability compared with rehabilitation center-based CR. However, there are also certain issues with home-based CR: (1) low participation and poor compliance of patients in exercising at home by themselves, (2) difficulty in monitoring exercise outcomes and injuries, (3) difficulty in making real-time adjustments to exercise prescriptions based on feedback from the patient's health status, and (4) difficulty in the real-time monitoring of abnormalities and risks in the CR process and daily activities of patients, as well as the lack of an early warning mechanism for the risks. All these problems seriously affect the rehabilitation outcome of patients with chronic HF.

**Fig. 2:**
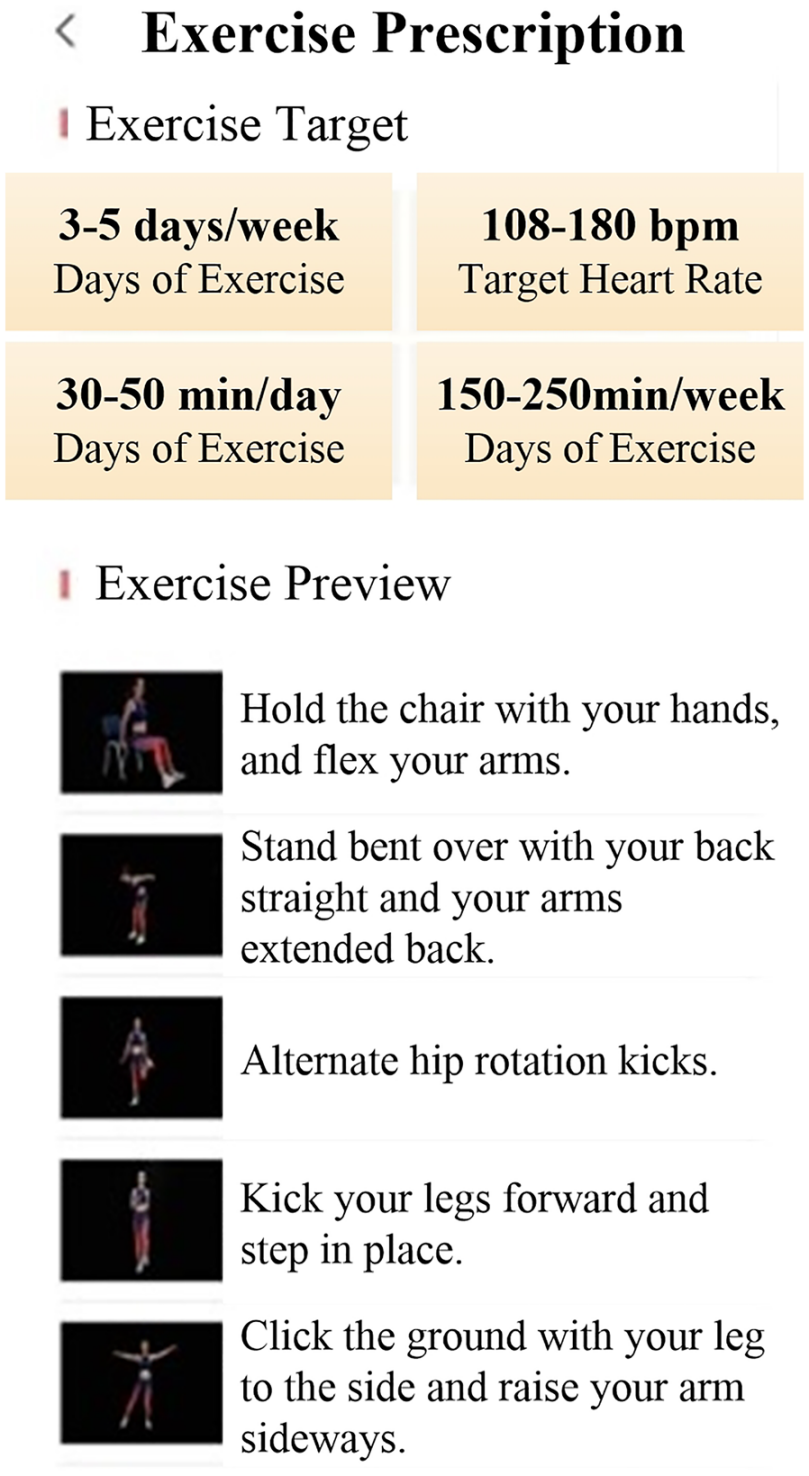
Exercise prescription

**Fig. 3:**
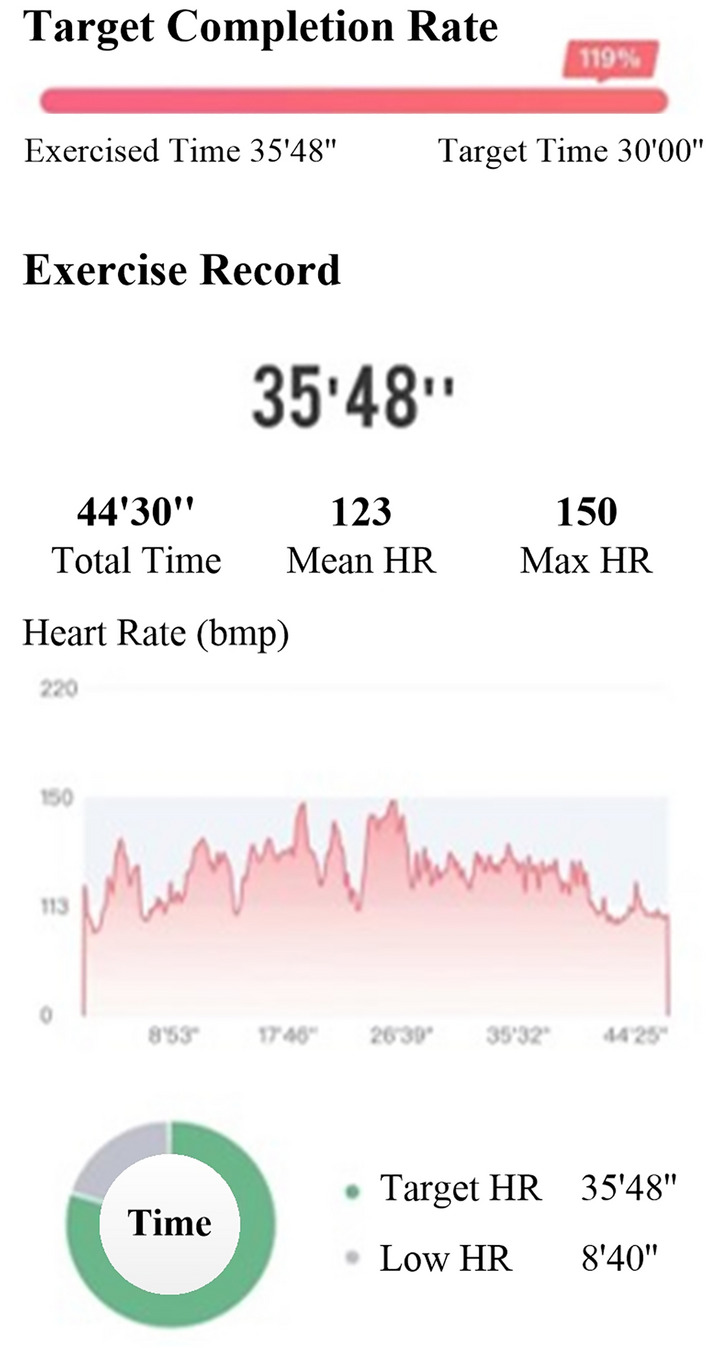
Monitoring information

**Fig. 4:**
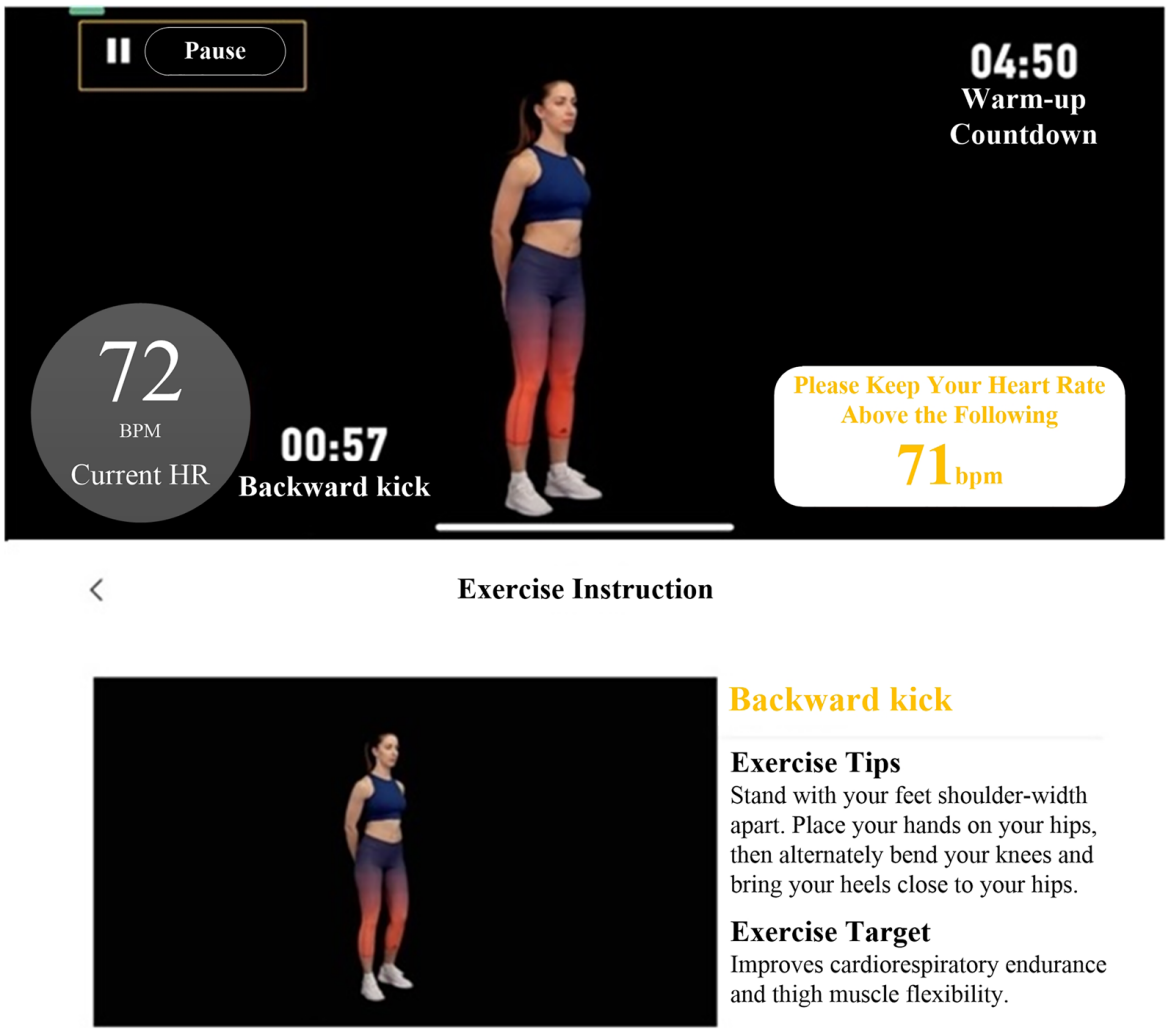
Guidance materials

In recent years, many guidelines and expert consensus [9, 13, 20] have recommended the incorporation of digital technologies and products into CR. Remote monitoring, rehabilitation guidance, and information feedback are used to provide patients with accurate home-based CR management and improve their compliance and rehabilitation outcomes. The results of many studies [26–30] have shown that CR with remote monitoring or telemonitoring is significantly better than conventional CR. Digital therapeutics (DTx), as defined by the *DTx Value Assessment & Integration Guide* [31]*,* is based on evidence-based medicine and uses digital means for data collection and indicator monitoring to control and optimize the treatment, management, and prevention of disease. Several countries have classified DTx as novel medical devices, and DTx and related products must pass clinical testing to obtain regulatory approval. The first DTx prescription was approved by the Food and Drug Administration in 2017. DTx has been applied to disease treatment, rehabilitation, prevention, and patient management in various fields, such as cardiovascular, endocrine, orthopedic, ophthalmological, neurological, and psychological. The results of several randomized controlled trials [32–41] showed that patients receiving DTx-based interventions had a higher level of health improvement and disease symptom relief and had significantly better psychological status and quality of life than control patients. In addition, DTx can enhance doctor-patient communication and play an active role in the management of disease risk factors, reduction of healthcare costs, and implementation of precision medicine [15, 42–44]. In 2019, the COVID-19 pandemic created barriers to face-to-face contact between physicians and patients, resulting in difficulties in providing offline medical care services. In this context, DTx has gradually gained the attention of doctors and patients and is now their preference. In 2020, the *ShuKang*™ (Recovery Plus Inc., China) App was approved for marketing by the National Medical Products Administration (NMPA) in China. The user interface is shown in Figs. 2–4. Fig. 2. Exercise prescription shows the personalized exercise prescription, including target heart rate, exercise movements, and exercise duration. Fig. 3. Monitoring information shows the monitoring information, including the exercise records and physiological indicators during exercise. Fig. 4. Guidance materials shows a video of the exercise movement instruction video for CR.

To enhance patient trust and acceptance, as well as ensure patient safety, the design of DTx should be based on randomized controlled trials and real-world evidence using an evidence-based medicine approach [45, 46]. Research and analysis of DTx should not only test its clinical effectiveness and safety from a medical perspective but should also assess its economic and social benefits from the perspective of health economics. However, current research on DTx is at the stage of clinical trial effect assessment, application prospect, and feasibility analysis, and there is less research on the cost-effectiveness analysis. Therefore, a comprehensive and multidimensional evaluation of DTx is imperative. In summary, we collected and reviewed hospital electronic medical record information, clinical trial research literature, health economics research literature, and other real-world data and built a model using assessment methods of health economics to analyze the potential cost-effectiveness of DTx applied to home-based CR for patients with chronic HF. From the perspective of medical and health decision-makers, the economic value of DTx is evaluated prospectively to provide a basis and reference for the application decision and promotion of DTx.

## Methods

To evaluate the effectiveness and economics of DTx for the home-based CR of patients with chronic HF, we are conducting a randomized controlled trial (ChiCTR2200060810), which is ongoing. This study focused on the methodological perspective of health economics and aimed to provide a prospective economic evaluation of DTx. Therefore, we selected real-world data hospital electronic medical record data, published research literature, and public website information to validate the constructed models and evaluation methods.

The Markov model is a well-established decision analysis model that takes relevant clinical data, costs, and health utilities as inputs, and the model outputs results, such as expected health states and treatment outcomes. Simulated objects enter the corresponding health state (stable, improving, relapse or deterioration, etc.) in the next cycle according to the state-transfer probability, and the expected outcome over the length of the simulation is assessed [47]. Based on the pathogenesis of HF and with reference to the relevant health economics evaluation literature, we used Markov models to simulate the rehabilitation effects of conventional home-based CR and DTx for home-based CR in patients with chronic HF in mainland China. Therefore, we assessed the potential role and value of DTx in CR in patients with chronic HF. The patients were divided into DTx for home-based CR (DT group) and conventional home-based CR (CH group) groups. All patients underwent home-based CR and received care guided by a multidisciplinary management program for HF according to the recommendations of *Rehabilitation Guidelines for Heart Failure*. Patients in the DT group were managed with the addition of DTx.

### Interventions

Patients were randomized in a 1:1 ratio into the intervention and control groups using a computer random number table. The specifications are as follows.

**Fig. 5:**
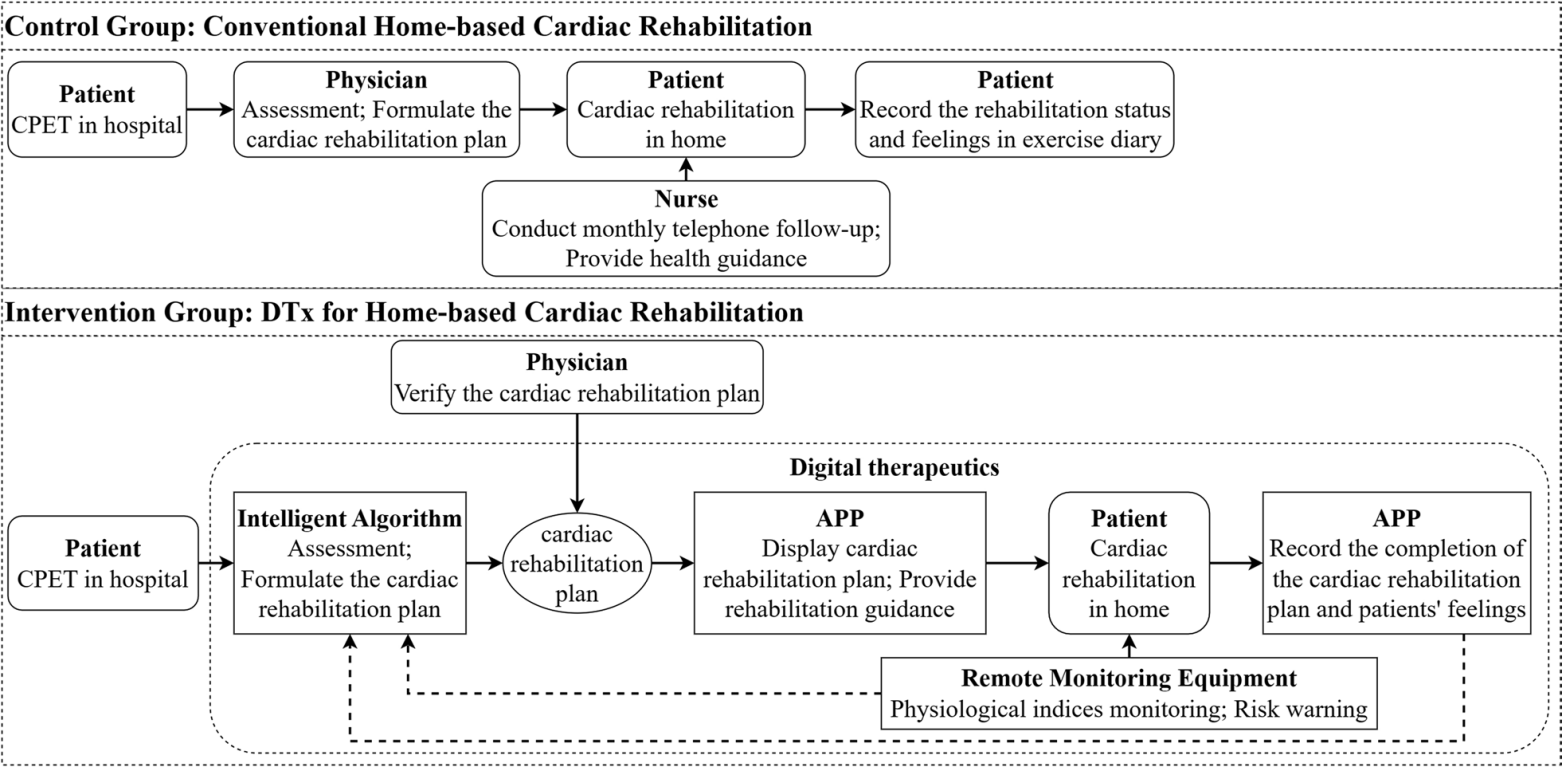
Interventions

In the control group (CH group), routine home-based CR management was provided, including in-hospital assessment and education and out-of-hospital exercise rehabilitation for 12 weeks (Fig. 5. Interventions). During the intervention, routine health guidance, including drugs, diet, activities, rest, and disease-related precautions, was provided by the nurses, and rehabilitation manuals were distributed. The patients were followed up monthly by telephone to determine their rehabilitation status. The patients underwent cardiopulmonary exercise testing (CPET). Exercise prescriptions were formulated according to the results of CPET following the FITTVP principle (F: exercise frequency; I: exercise intensity; T: exercise time; T: exercise type; V: exercise volume; and P: exercise progression) [20]. The specifics are as follows. (1) Aerobic exercise was predominant. (2) Low-to-moderate-intensity exercise, reflected by the patient's heart rate during exercise, was prescribed. (3) Exercise was prescribed 3–5 times per week. (4) The cumulative time to reach the target heart rate in each exercise was ≥ 30 min, and the time of exercising was ≥ 150 min weekly. The target heart rate (heart rate_target_) was defined as (heart rate_max_-heart rate_rest_)–(40–60)% + heart rate_rest_, and the alarm heart rate (heart rate_alarm_) was heart rate_target_ + 20 bpm [56]. The patients' rehabilitation status and feelings were recorded in their exercise diaries. In addition, relevant supervision, including medication reminders and diet and lifestyle guidelines, was undertaken.

In the intervention group (DT group), DTx for home-based CR management was offered for 12 weeks based on routine rehabilitation management (Fig. 5. Interventions).

The specifics are as follows. (1) Patients got CPET in the hospital. Based on the assessment results, an intelligent algorithm for Digital Therapeutics was used to formulate exercise prescriptions. Their contents also follow the FITTVP principle. (2) The exercise prescription was sent to the patient's mobile phone through the *Shukang* App (Recovery Plus Inc., China) after verification by the physician, and both the heart rate_target_ and heart rate_alarm_ were set. (3) Patients wore wearable and portable devices such as heart rate bands. The heart rate of the patient during exercise was monitored in real time, and an alarm was raised in case of an abnormality. Moreover, heart rate during daily life activities and exercise was monitored, and abnormal signals were captured in real time and transmitted to the monitoring center through the patient's mobile phone so that the rehabilitation team could make a judgment and promptly give its opinion on diagnosis and treatment. (4) The total exercise time, effective time (heart rate_target_ maintained) of exercise, time to recover to resting heart rate, and subjective feeling about the exercise were recorded by the app, based on which the professional rehabilitation team adjusted the exercise prescription and answered questions one to one online.

### Model development

**Fig. 6:**
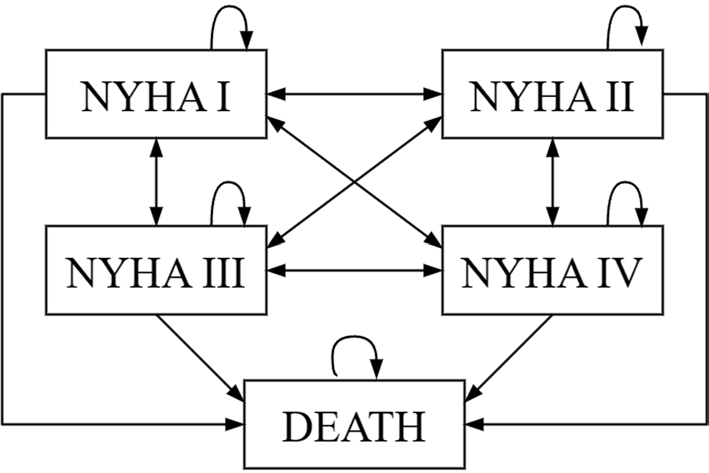
State transfer of Markov model

As shown in Fig. 6. State transfer of Markov model, five states were set: NYHA I, NYHA II, NYHA III, NYHA IV, and death. Based on the disease progression of HF and related studies, the simulation time of the Markov model was set to 10 years or patient death (whichever occurred first), with a cycle period of 1 month for a total of 120 cycle periods. Initially, the patients entered the cohort in one of the NYHA I-IV states and moved to another state or maintained the original state with a corresponding probability in each cycle. Patients could only be in one state during the same cycle.

The incremental cost-effectiveness ratio (ICER) was used as the analysis result to compare the difference in cost-effectiveness between the DT and CH groups. The ICER was calculated as follows: The willingness-to-pay (WTP) threshold was set at 85,698 CNY (one time of China's GDP per capita in 2022).

$$ICER=\frac{{COST}_{DT}-{COST}_{UC}}{{QALY}_{DT}-{QALY}_{UC}}$$

### Model inputs

The outcomes measured and collected in this study are demographic data, medical cost data and health utility data. Among them, the health utility data includes quality-adjusted life years (QALYs), the probability of state transition between NYHA I-IV, and the risk ratio of hospitalization for patients.

All data were obtained from real-world sources (including clinical trial data, observational study data, health insurance data, and national survey data, etc.), hospital electronic medical record data (including outpatient and inpatient medical record data, health checkup data, medical tests and imaging data, electronic prescription, and diagnostic certificate data, etc.), published research literature, and public website information. The search period for the literature was from 2010 to 2022, and the databases used for the literature search included PubMed, Medline, Web of Science, and CNKI. For the real-world data collected, data from randomized controlled clinical trials in mainland China and research data from health economics assessments were used as the preferred basis for the model input parameters. The Markov model inputs were the number and proportion of patients with initial states NYHAI-IV, state transition probability matrix, cost data, and health utility values.

Patient clinical and demographic data were all obtained from the electronic medical record database of a tertiary hospital in Jiangsu Province, China, as shown in Table 1. The period of data was 2019–2021, and the data included 2315 patients (sample size n = 2315). Based on the data obtained, the total number of patients in the Markov model was set to 2315, and the number of patients with NYHA grades I-IV and death status at baseline were 1795, 172, 280, 68, and 0, respectively.

**Table 1:** Clinical data and demographics of patients

Variable	Numerical value
Mean age (years)	62.86
Sex (case)	
Male	1574 (67.99%)
Female	741 (32.01%)
NYHA functional class (case)	
I	1795 (77.54%)
II	172 (7.43%)
III	280 (12.09%)
IV	68 (2.94%)
Average number of days in hospital (days)	
NYHA I	6.25
NYHA II	10.16
NYHA III	13.57
NYHA IV	12.91
Medication (case)	
B-blocker	1828 (78.96%)
Diuretic	1987 (85.83%)
ACE inhibitor	489 (21.12%)
Angiotensin Receptor inhibitor	1567 (67.69%)
Digitalis	568 (24.54%)
Mineralocorticoid receptor antagonist	1670 (72.14%)
Sacubitril valsartan	874 (37.75%)
Comorbidities (case)	
Hypertension	1456 (62.89%)
Diabetes mellitus	678 (29.29%)
Atrial fibrillation	436 (18.83%)
Coronary heart disease	1034 (44.67%)
Cardiac valve disease	409 (17.67%)
Cardiomyopathy	103 (4.45%)

In this study, a cost-effectiveness analysis was performed from the perspective of healthcare decision-makers; the items, data, and data sources involved in costing are shown in Table 2. The discount rates were all calculated at 3% per year. Cost accounting for health economics assessments should include direct medical, direct non-medical, and indirect costs [48]. Direct medical costs refer to the costs of medical resources consumed for a certain treatment option. Direct non-medical costs are costs for resources other than those directly consumed by the patient to seek medical services, while indirect costs are the loss of productivity by the patient and family as a result of the disease. Direct medical costs are the average per-patient hospitalization costs for HF patients and the cost of DTx for the DT group. The data source for hospitalization costs was the electronic medical record database of a tertiary hospital in Jiangsu Province, China, which spanned from 2019 to 2021 and involved 2315 patients (sample size n = 2315). The annual membership fee of the *Keep* app in 2022 was used as a reference for the cost of DTx, which was 180 CNY/year. This service included developing exercise plans and providing exercise guidance. The *2021 China Smart Fitness Industry Research Report* [49] showed that *Keep* is a leading application in China that provides exercise and fitness guidance as well as smart monitoring. *Keep* makes exercise recommendations based on the user’s exercise capacity assessment and formulate a fitness exercise plan after it is reviewed by a coach. Moreover, exercise guidance is displayed via pictures, voices, and videos, and the user’s exercise trajectory and body indicators can be monitored using mobile phones, watches, and other mobile devices. Users can use the app to record and upload exercises and feelings, and the exercise plan can be adjusted based on feedback. Therefore, *Keep* is similar to DTx, in that it provides guidance and monitoring. In addition, the family doctor service program in Jiangsu Province, China [50], which charges 90 CNY/year per person, was used as a reference for the cost of DTx. Family doctors can provide online primary care consultation services to contracted patients.

**Table 2:** Costs

	Cost	NYHA I	NYHA II	NYHA III	NYHA IV	Data source
CH group	Direct medical costs (CNY/visit) Direct non-medical costs (CNY/year) Indirect costs (CNY/year)	35,587.73 1012.25 45,720	39,014.21 2032.93 21,342.13	53,577.65 2040.54 31,370.21	59,547.03 5333 23,085.54	Electronic medical records [51] [51]
DT group	Direct medical costs (CNY/visit) Direct non-medical costs (CNY/year) Indirect costs (CNY/year) Cost of DTx (CNY/year)	35,587.73 1012.25 45,720 180	39,014.21 2032.93 21,342.13 180	53,577.65 2040.54 31,370.21 180	59,547.03 5333 23,085.54 180	Electronic medical records [51] [51] Official website

The probability of state transition between NYHA I-IV is shown in Table 3, and the risk ratio of hospitalization for patients in DT group versus that of patients in CH group is shown in Table 4 Risk ratio of hospitalization for patients of DT group versus patients of CH group, with a study [51] used as a reference for the data. This study calculated the probability of transition between NYHA I-IV and the probability of hospitalization based on the UK National Hospital Episode Statistics (which contained details of all inpatient, outpatient, and emergency visits to National Health Service hospitals in the UK). Due to the lack of trials on DTx for home-based CR for patients with chronic HF, data from the trial on home-based CR under telemonitoring were used. In home-based CR, under telemonitoring, the patients' physiological indicators during daily life and exercise are monitored using portable mobile devices. When the indicators are abnormal, the doctor or rehabilitation team takes immediate action to prevent risk and danger. DTx is a more comprehensive and precise intervention based on remote monitoring. It provides control, feedback, and optimization of the entire home-based cardiac process, which provides more precise management for patients and effectively improves outcomes and adherence. Compared to telemonitoring, DTx for home-based CR has better outcomes, better patient health status, and a lower risk of hospitalization. Therefore, we used data from a study of Home Cardiac Rehabilitation in Chronic Heart Failure Patients with Remote Monitoring trial to estimate the effectiveness of digital-based therapy for home-based CR. Therefore, data from a study on home-based CR using telemonitoring in patients with chronic HF was used to estimate the effect of DTx for home-based CR.

**Table 3:** Monthly transition matrix of Markov model and probability of monthly hospitalization

CH group	Probability of state transfer					Probability of hospitalization	Data source
	NYHA I	NYHA II	NYHA III	NYHA IV	Death		
NYHA I	0.981	0.004	0	0	0.015	0.004	[52]
NYHA II	0.068	0.872	0.017	0	0.043	0.020	[52]
NYHA III	0.004	0.094	0.777	0.041	0.084	0.053	[52]
NYHA IV	0	0.006	0.095	0.777	0.122	0.085	[52]
Death	0	0	0	0	1	0	[52]
DT group	Probability of state transfer					Probability of hospitalization	Data source
	NYHA I	NYHA II	NYHA III	NYHA IV	Death		
NYHA I	0.986	0.003	0	0	0.011	0.003	[52]
NYHA II	0.068	0.885	0.013	0	0.033	0.015	[52]
NYHA III	0.004	0.096	0.804	0.032	0.065	0.040	[52]
NYHA IV	0	0.006	0.098	0.801	0.094	0.064	[52]
Death	0	0	0	0	1	0	[52]

**Table 4:** Risk ratio of hospitalization for patients of DT group versus patients of CH group

Type	Risk ratio of hospitalization	Data source
NYHA I	0.75	[52]
NYHA II	0.75	[52]
NYHA III	0.7547	[52]
NYHA IV	0.7529	[52]

The health utility data were the quality-adjusted life years (QALYs) of the patients, as shown in Table 5. Health utility values, with reference data from a study [26]. This study used real-world data to assess the cost-effectiveness of rehabilitation management in patients with chronic HF. The discount rate for the QALYs was set at 3% per year.

**Table 5:** Health utility values

Type	Mean	Variance	Data source
NYHA I	0.87976	0.00827	[26]
NYHA II	0.71178	0.00944	[26]
NYHA III	0.61405	0.01349	[26]
NYHA IV	0.49228	0.03032	[26]

Two-way and one-way sensitivity analyses were performed on the model input parameters to test the degree of influence of each parameter on the model output results. The ranges and distribution of the parameter variations are listed in Table 6. The effect of direct medical cost on the model results was tested using the inpatient costs of patients with HF at another tertiary hospital. Data were obtained from the electronic medical record database of a tertiary hospital in Sichuan Province, China. The data spanned from 2018 to 2022 and included 1265 patients (sample size, n = 1265). Moreover, the relevant literature was referred to for the selection of the range of parameter changes. “Heart failure,” “rehabilitation,” and “cost-effectiveness analysis” were used as keywords to search databases, including PubMed, Medline, Web of Science, and CNKI, for literature published between 2010 and 2022 to ultimately identify five research articles as references for the range of parameter changes. Probabilistic sensitivity analyses were conducted using Monte Carlo simulations to test the cost-effectiveness probability for the DT and CH groups at different WTP thresholds. Monte Carlo simulations were performed 10,000 times with reference to the study [27], and the parameter distributions are listed in Table 6. Range and distribution of parameter changes. The probabilities of each strategy to be accepted as cost-effectiveness in the 10,000 Monte Carlo simulations were determined against the variation of the WTP threshold in the acceptability curve. According to the recommendation of the World Health Organization [53], the program is considered highly cost-effective when the ICER is less than the GDP per capita. The GDP per capita in China for 2022 (85,698 CNY) was used as the WTP threshold.

**Table 6:** Range and distribution of parameter changes

Item	Range of parameter changes	Parameter distribution	Data source
Direct medical costs		Gamma	Electronic medical records, [54]
NYHA I	6942.58-42,705.28		
NYHA II	26,095.71–46,817.06		
NYHA III	42,862.12–64,293.19		
NYHA IV	42,488.95–71,456.43		
Direct non-medical costs		Gamma	[54]
NYHA I	36,576–54,864		
NYHA II	17,073.7–25,610.56		
NYHA III	25,096.17–37,644.25		
NYHA IV	18,468.43–27,702.65		
Indirect costs		Gamma	[54]
NYHA I	809.8–1214.7		
NYHA II	1626.34–2439.52		
NYHA III	1632.43–2448.65		
NYHA IV	4266.4–6399.6		
Cost of DTx	144–216	Gamma	[28]
Health utility	0.66–0.96	Beta	[22, 54]
Hospitalization risk ratio	0.36–0.85	Lognormal	[22, 54]

## Results

### Cost-effectiveness analysis

The expected incremental QALY and costs for the DT and CH groups are presented in Table 7. The per capita cost for the CH group was 38,442.11 CNY/year, and the per capita QALY per year was 0.7196. The per capita cost for the DT group was 42,300.26 CNY/year, and the per capita QALY per year was 0.81687. Compared to the CH group, the per capita incremental QALY per year for patients in the DT group was 0.09727, with an additional cost of 3858.15 CNY/year and an annual per capita ICER of 39,663.5 CNY/QALY, which was below the WTP threshold of 85,698 CNY (China’s GDP per capita in 2022).

**Table 7:** Results of the cost-effectiveness analysis

Group	Cost (CNY)	Incremental cost (CNY)	QALYs	Incremental QALY	ICER (CNY/QALY)
CH group	38,442.11	–	0.71959	–	–
DT group	42,300.26	3857.86	0.81686	0.09727	39,661.32

**Fig. 7:**
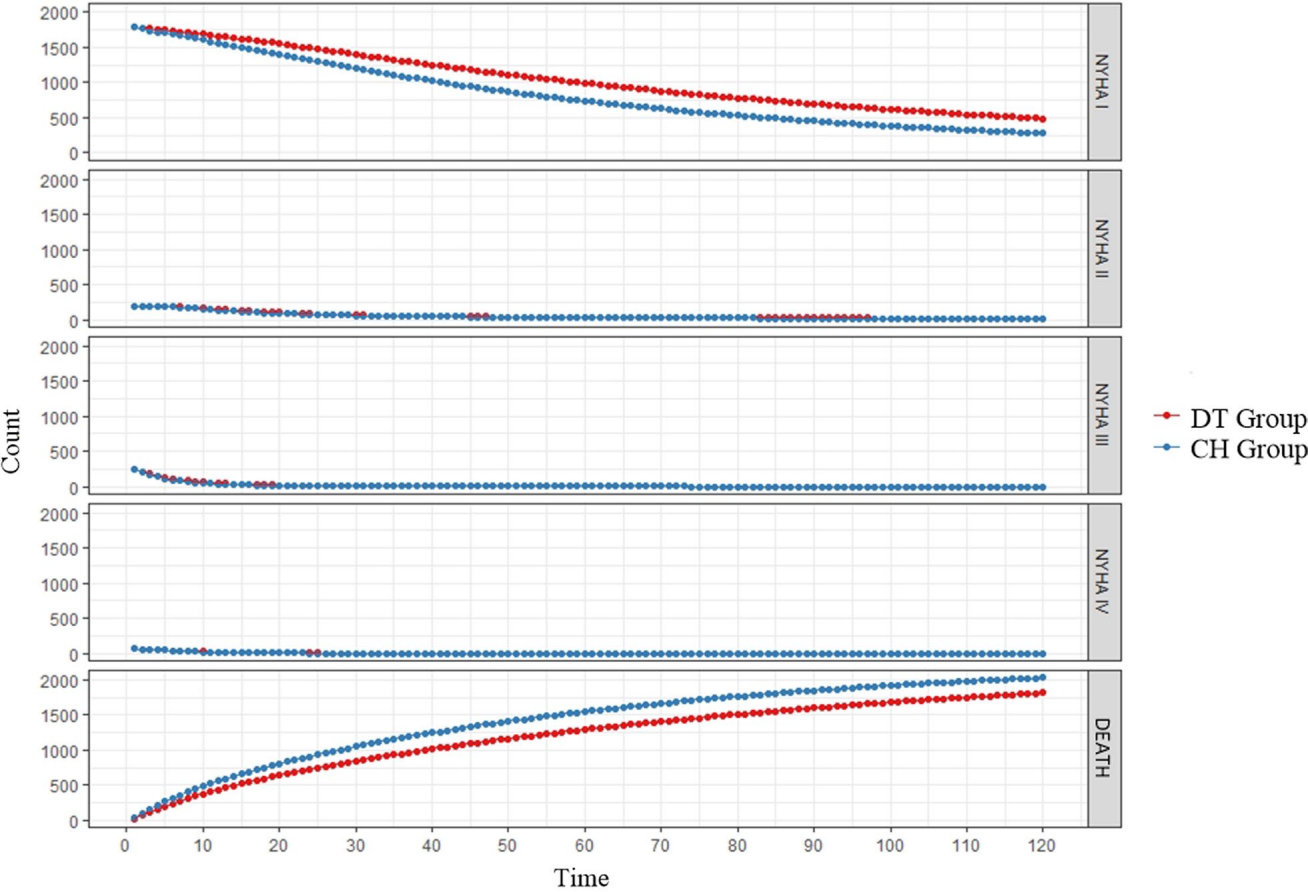
Health state transfer

The modeling results of the patients' health statuses over 10 years are shown in Fig. 7 Health state transfer.

Compared with the CH group, the health status of patients in the DT group was more stable, and there were fewer deaths in the DT group as the modeling period was extended. Therefore, the health status of patients in the DT group was significantly better than that of patients in the CH group.

### Sensitivity analysis

#### Single-factor sensitivity analysis

**Fig. 8:**
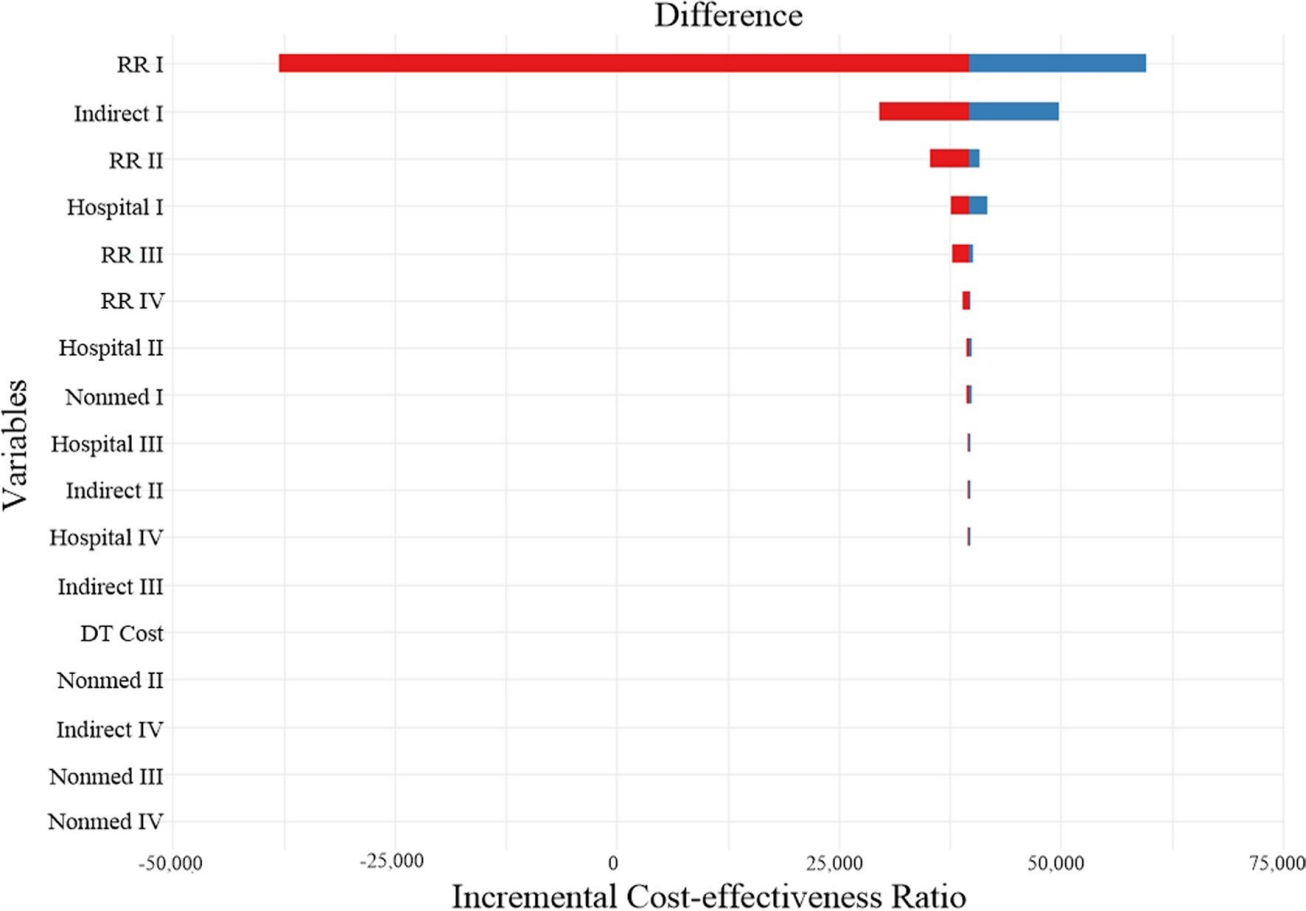
Tornado chart of sensitivity analysis

A tornado chart of the sensitivity analysis is shown in Fig. 8.

The results showed that the risk ratio for hospitalization due to HF had the greatest impact on the ICER. The remaining parameters were ranked according to their impact on the ICER: indirect costs, direct medical costs (inpatient costs), direct non-medical costs, and DTx costs.

#### Probabilistic sensitivity analysis

**Fig. 9:**
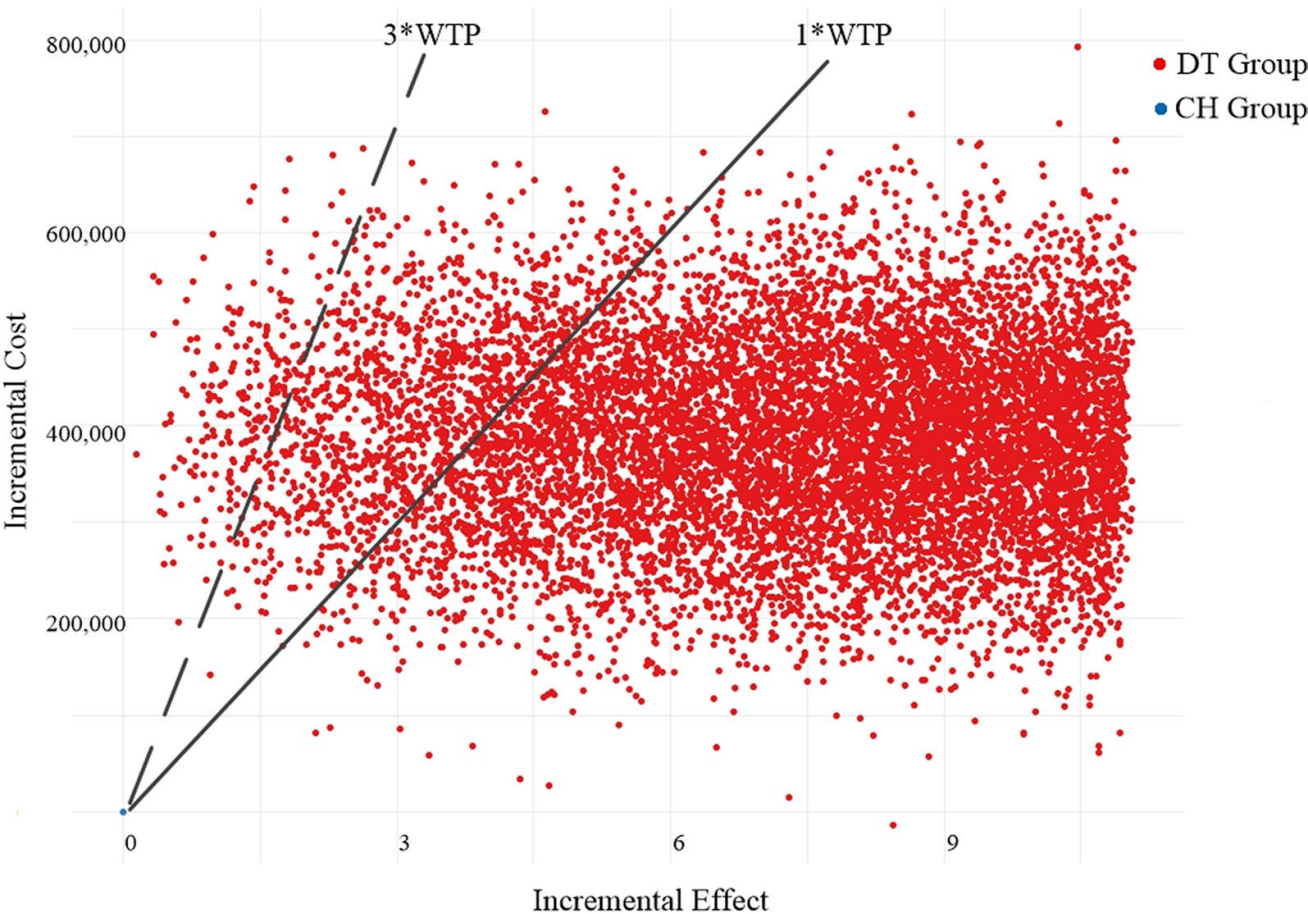
Scatter plot of ICER

**Fig. 10:**
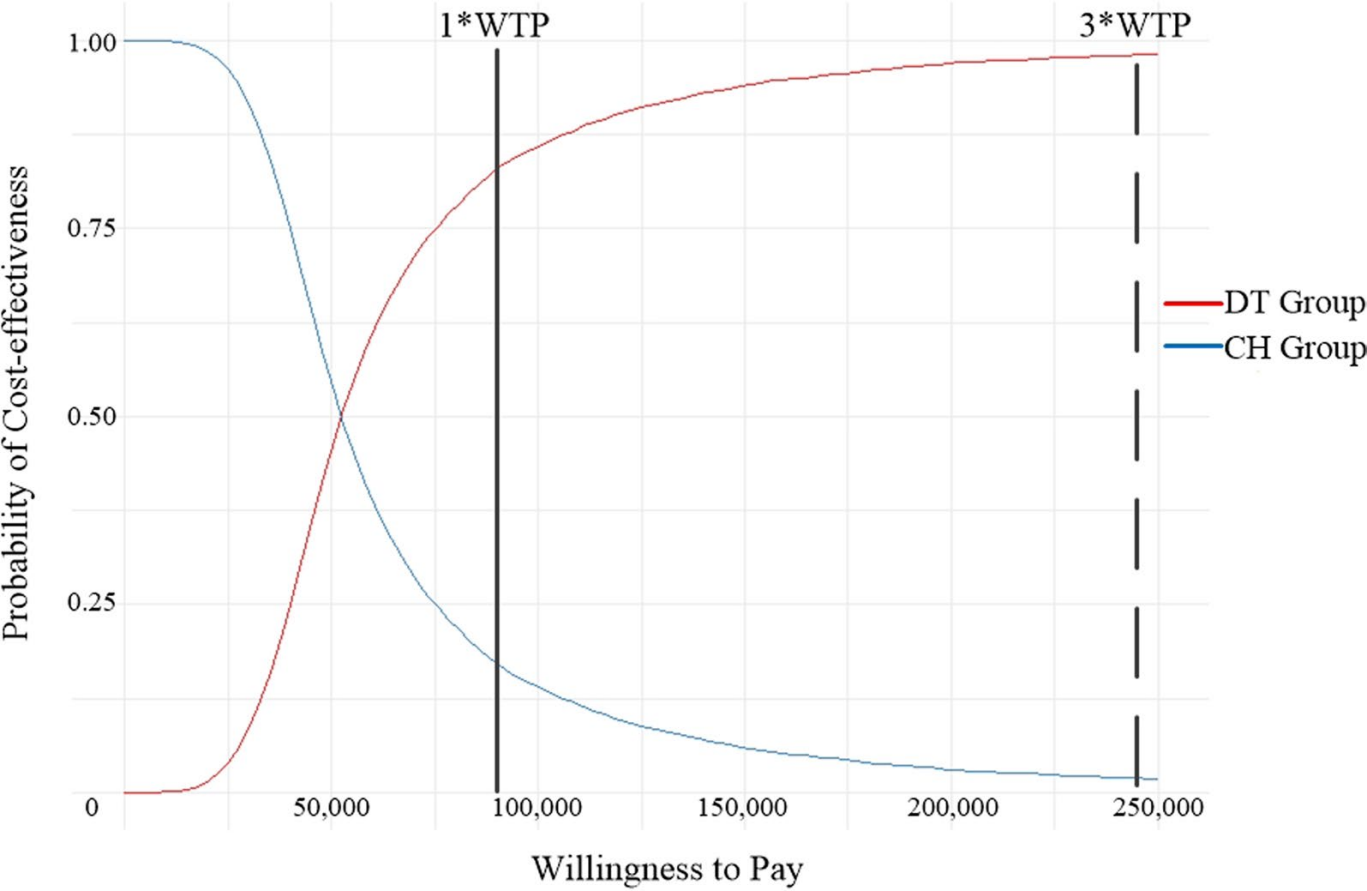
Cost-effectiveness acceptability curve

According to the parameter distribution of the relevant items o the model inputs, the Markov model was subjected to probabilistic sensitivity analysis using Monte Carlo simulation to obtain the ICER scatter plot (REF Figure_9 \h \* MERGEFORMAT Fig. 9) and the cost-effectiveness acceptability curve (REF Figure_10 \h \* MERGEFORMAT Fig. 10). As shown in (REF Figure_9 \h \* MERGEFORMAT Fig. 9), the vast majority of scattered points fell below the threshold line when using 1x and 3x of China's GDP per capita in 2022 as the WTP threshold, indicating that the DT group was more likely to be cost-effective than the CH group. As shown in REF Figure_10 \h \* MERGEFORMAT Figure 10, when using 85,698 CNY (1x 2022 Chinese GDP per capita) as the WTP threshold, the DT group had an 82.7% probability of being cost-effective. Using 257,094 CNY (3x 2022 Chinese GDP per capita) as the WTP threshold, the DT group had a 96.6% probability of cost-effectiveness. Meanwhile, the probability of the DT group having the advantage of being cost-effective increased with an increase in the WTP threshold.

## Discussion

Innovative digital technologies and products will be an important development trend in the prevention, treatment, and management of diseases as well as healthcare services in the future. As a frontier of innovation, DTx plays an increasingly prominent role in the global healthcare industry, pioneering a new approach to medical intervention and disease management. The flourishing development of DTx has complemented and optimized traditional diagnostic and therapeutic tools that greatly inform clinical intervention decision-making and bring significant economic and social benefits to patients, physicians, and healthcare decision-makers. The use of DTx in CR has become increasingly widespread, especially after the outbreak of the COVID-19 pandemic, when patients faced difficulties in accessing care, as it was difficult to maintain offline face-to-face physician–patient contact, communication, and rehabilitation care, as well as guidance to reduce the risk of COVID-19 transmission. Given this background, an increasing number of patients with chronic HF have opted DTx for home-based CR. This study collected and reviewed real-world data and built a model using health economics assessment methods to analyze the potential cost-effectiveness of DTx in home-based CR for patients with chronic HF. From the perspective of medical and health decision-makers, the economic value of DTx is evaluated prospectively, providing a scientific basis and reference for the formulation of treatment plans for chronic HF, the analysis of patient compliance, and the decision to promote DTx. Based on the study results, it was found that DTx can be of great utility to patients, doctors, and healthcare decision-makers.

The advantages of DTx for patients are as follows: (1) The clinical outcomes and quality of life have improved significantly. Studies [42–44] have demonstrated the safety of DTx, which plays a positive role in improving patient compliance, enhancing doctor-patient communication, and aiding in the management of disease risk factors. DTx enables real-time remote monitoring, which reduces the risk to patients' daily lives and home-based CR. DTx provides precise medical care, including clinically validated personalized treatment plans. In addition, DTx helps patients delay disease progression and reduce complications through self-management, improving the quality of life with the disease and reducing the panic of patients who are at a loss as their disease evolves. In this study, we constructed a Markov model to simulate changes in the health status of patients with chronic HF over a 10-year period. The results showed that the health status of patients who underwent DTx for home-based CR were significantly better than that of patients who underwent conventional home-based CR. Moreover, patients on DTx for home-based CR were better able to maintain a stable health status as the cycle was extended. Furthermore, the mortality rate of the patients who underwent DTx for home-based CR was lower during the simulated cycle. Therefore, we conclude that DTx is more effective for home-based CR than conventional home-based CR. With DTx intervention and management, patients may achieve a more stable and better health status. (2) Cost savings for medical services. The results of this study showed that the additional annual per capita cost for patients in the DT group was 3,858.15 CNY/year compared with the CH group, and the annual per capita ICER was 39,663.5 CNY /QALY, which was below the WTP threshold of 85,698 CNY (GDP per capita in China in 2022). Therefore, DTx for home-based CR is extremely cost-effective. In the sensitivity analysis, the factors were ranked according to their impact on the ICER, which were indirect costs, direct medical costs (inpatient costs), direct non-medical costs, and DTx costs. Thus, DTx is more economical than conventional home-based CR and helps reduce patients' medical expenses. Indirect costs include the loss of productivity to the patient and family members due to illness or death, in addition to the cost of hiring a companion for the patient [48]. First, with the intervention and management of DTx, patients with chronic HF have better outcomes for home-based CR, resulting in less time being missed from work and lost productivity. Second, DTx provides real-time monitoring and rehabilitation guidance, reducing the patient's dependence on a companion and the cost of hiring a companion. Finally, patients can exercise at home with the guidance and assistance of DTx, eliminating the need to go to a rehabilitation center for CR, which reduces the cost of transportation and time and avoids the hassle of traveling to and from medical appointments. In addition, DTx is generally less expensive than rehabilitation. The cost of DTx had a small impact on outcomes, and the results suggested that most patients accepted the cost of DTx within a reasonable range. (3) Improving health values. First, DTx develops personalized rehabilitation programs and makes dynamic and optimal adjustments based on the collected data and feedback, thus enabling the monitoring, control, and optimization of the entire patient management process, thereby enhancing patient satisfaction and experience and improving adherence. Studies [27, 29] have shown that patient compliance with CR increased by 80% with the intervention of DTx. Second, DTx provides targeted rehabilitation programs and guidance that effectively improve rehabilitation outcomes, help patients delay disease progression, and reduce complications through self-management. Studies [27, 55] have shown that the probabilities of hospitalization for HF and all-cause mortality were 50% and 81%, respectively, for patients on DTx for home-based CR compared with patients who did not use the program. Results from several randomized controlled trials of studies [32–41] showed that patients with DTx interventions had higher levels of health improvement, disease symptom relief, and reduced panic or confusion during disease progression. Thus, DTx improved patients’ quality of life with higher psychological status (mood, health beliefs, etc.) and satisfaction, significantly increasing their health value.

The advantages of DTx for clinicians are as follows: (1) Providing reliable clinical advice to clinicians and care teams, (2) uninterrupted monitoring and assessment of patient's health status to reduce the risk of adverse events such as side effects and comorbidities, and (3) real-world data can be obtained from DTx for clinical research, such as assessing the impact of therapies on treatment goals and optimizing, adjusting, recommending, enhancing, or reducing treatment intensity.

For healthcare decision-makers, the advantages of DTx are as follows: (1) Improving healthcare accessibility. DTx enables the migration of treatment scenarios and the transformation of the subject of the implemented interventions, increasing the accessibility of disease treatment tools. (2) Integration of DTx with home and community health services to expand the ability of patients to access active clinical care in traditional settings and beyond (e.g., home care, telecare, digital healthcare). (3) The ability to provide long-term care for patients (including those with chronic conditions, older adults, persons with disabilities, low-income, and patients who require companion care, etc.) with innovative optional treatment options. (4) Provide technical support services to patients, caregivers, and other end users to alleviate the lack of medical resources in remote areas such as rural areas.

### Limitations

Clinical data from randomized controlled trials supporting health economic assessments are lacking. Therefore, this study focused on the methodological perspective of health economics and aimed to provide a prospective economic evaluation of DTx for the home-based CR of patients with chronic HF. Currently, there is an ongoing randomized controlled trial of DTx for home-based CR of patients with chronic HF (the trial has been registered on the World Health Organization International Clinical Trials Registry Platform, registration number: ChiCTR2200060810). A comprehensive assessment of DTx involving multiple dimensions of clinical indicators and psychological and economic benefits will be conducted in the future.

### Conclusions

The results showed that the per capita cost of conventional home-based CR was 38,442.11 CNY /year, with an annual per capita QALY of 0.7196. The per capita cost of the DTx for home-based CR was 42,300.26 CNY /year, with an annual per capita QALY of 0.81687. The annual per-capita ICER was 39,663.5 CNY /QALY, which was below the WTP threshold of 85,698 CNY (China's GDP per capita in 2022). Therefore, DTx for home-based CR has certain advantages in terms of cost-effectiveness. From the perspective of healthcare decision-makers, DTx may be incorporated into home-based CR for chronic HF and may be a potentially valuable intervention.

## Data Availability

The data covered in this article are derived from electronic medical records, which have been approved by the First Affiliated Hospital of Nanjing Medical University.

## Abbreviations

CPET: Cardiopulmonary exercise testing
CR: Cardiac rehabilitation
COVID-19: Coronavirus disease 2019
DTx: Digital therapeutics
HF: Heart failure
ICER: Incremental cost-effectiveness ratio
QALYs: Quality-adjusted life years
WTP: Willingness-to-pay
