# Correction: Cost-effectiveness analysis of digital therapeutics for home-based cardiac rehabilitation for patients with chronic heart failure: model development and data analysis

**DOI:** 10.1186/s12962-024-00524-5

**Published:** 2024-02-26

**Authors:** Tianyi Liu, Yiyang Zhan, Silei Chen, Wenhong Zhang, Jian Jia

**Affiliations:** 1 School of Business, Nanjing University, 210093 Nanjing, China; 2 Departments of Geriatric Practice, The First Affiliated Hospital of Nanjing Medical University, 210029 Nanjing, China; 3 Medical School, Nanjing University, Nanjing, China; 4 National Institute of Healthcare Data Science, Nanjing University, Nanjing, China; 5 Departments of General Practice, The First Affiliated Hospital of Nanjing Medical University, Nanjing, China

Cost Effectiveness and Resource Allocation (2023)21:82

10.1186/s12962-023-00489-xIF:2.3Q3B4

Following publication of the original article, the authors flagged that the following acknowledgement of funding had been omitted from the Funding declaration: ‘funding and support from the National Natural Science Foundation of China (Grant No. 72072083)’.

The original article [1] has been corrected.

